# The Registry of Knowledge Translation Methods and Tools: a resource to support evidence-informed public health

**DOI:** 10.1007/s00038-013-0448-3

**Published:** 2013-02-08

**Authors:** Leslea Peirson, Cristina Catallo, Sunita Chera

**Affiliations:** 1 McMaster Evidence Review and Synthesis Centre, McMaster University, Downtown Centre, 50 Main St. E., 3rd Floor, Hamilton, ON Canada; 2 Daphne Cockwell School of Nursing, Ryerson University, Toronto, ON Canada; 3 National Collaborating Centre for Methods and Tools, McMaster University, Hamilton, ON Canada

**Keywords:** Knowledge translation, Public health, Methods, Tools, Registry

## Abstract

**Objectives:**

This paper examines the development of a globally accessible online Registry of Knowledge Translation Methods and Tools to support evidence-informed public health.

**Methods:**

A search strategy, screening and data extraction tools, and writing template were developed to find, assess, and summarize relevant methods and tools. An interactive website and searchable database were designed to house the registry. Formative evaluation was undertaken to inform refinements.

**Results:**

Over 43,000 citations were screened; almost 700 were full-text reviewed, 140 of which were included. By November 2012, 133 summaries were available. Between January 1 and November 30, 2012 over 32,945 visitors from more than 190 countries accessed the registry. Results from 286 surveys and 19 interviews indicated the registry is valued and useful, but would benefit from a more intuitive indexing system and refinements to the summaries. User stories and promotional activities help expand the reach and uptake of knowledge translation methods and tools in public health contexts.

**Conclusions:**

The National Collaborating Centre for Methods and Tools’ Registry of Methods and Tools is a unique and practical resource for public health decision makers worldwide.

## Introduction

In order to strengthen public health systems decision makers need the right type of research evidence at the right time. Strategies and resources that help decision makers access and utilize research evidence are crucial (Frank et al. 2007; Kiefer et al. 2005). Many countries promote public health core competencies which require proficiency in using research evidence to inform decision making [e.g., Canada (Public Health Agency of Canada 2010); United Kingdom (Public Health Resource Unit 2008); United States (Public Health Foundation 2010); Australia (Australian Network of Public Health Institutions 2009)], however, the public health workforce generally lacks the skills and resources to accomplish these standards (Bowen et al. 2009; Peirson et al. 2012). Inconsistent skill capacity for evidence-informed decision making combined with limited resources make effective implementation of evidence-informed public health a formidable task. Even so, some recent initiatives have tackled these challenges in attempts to build individual and organizational capacity to better align public health programs and policies with the best available scientific evidence (e.g., Dobbins et al. 2004, 2009b; Peirson et al. 2012). However, more can be done to support such efforts and to facilitate and improve evidence-informed decision making throughout public health systems.

In 2007, the National Collaborating Centre for Methods and Tools (NCCMT) was officially launched as a part of the Government of Canada’s commitment to renew and strengthen public health across the country (Medlar et al. 2006). The mandate of the NCCMT is to improve public health stakeholders’ access to and use of knowledge translation (KT) methods and tools to support evidence-informed public health (Frank et al. 2007). As defined by the Canadian Institutes of Health Research (CIHR), KT involves dynamic and iterative processes of synthesizing, disseminating, exchanging and applying knowledge for the purposes of ensuring effective health services and products, enhancing the health care system and promoting health (Canadian Institutes of Health Research 2012). An environmental scan, key informant interviews and surveys conducted to inform the NCCMT’s work plans discerned the need to identify KT methods and tools relevant for public health in Canada and to facilitate quick and easy access to these resources by decision makers (Ciliska et al. 2006). In response, one of the first projects undertaken by the NCCMT was to create a searchable database of these resources and freely disseminate this information through its publicly accessible website.

The NCCMT’s Registry of Methods and Tools (http://www.nccmt.ca/registry/index-eng.html) was conceived and constructed as an interactive and expanding online database of resources to support public health decision makers to incorporate KT in public health practice. These resources include both methods (standardized processes, regular and systematic approaches, or sets of organized steps) and tools (standardized products such as instruments, surveys and checklists) that facilitate access to and use of research evidence in decision making and reflect the four types of KT activities as defined by CIHR (synthesis, dissemination, exchange and application). In this paper, we describe: how the registry was developed and initially populated as a public health resource; the methods and results of a recent systematic literature search for KT resources; the current contents, features and users of the registry website; and ongoing efforts to evaluate and enhance the registry. We conclude by discussing the relevance of the NCCMT’s Registry of Methods and Tools as a unique, sustainable and internationally relevant resource for promoting evidence-informed public health.

## Methods

### Registry development

The development of the registry began in fall 2007 by establishing an advisory group comprised of 18 national and international public health policy and program decision makers, and KT researchers. Work plans, processes and tools for building the database and website were developed with input from this group. In order to locate appropriate KT methods and tools, a comprehensive search strategy was developed that included both published and unpublished literature. To determine the relevance of identified resources an inclusion-screening tool was developed that contains three key assessment criteria: (1) describes a KT activity, (2) contains a method or a tool, and (3) can be used and/or adapted for use in Canadian public health contexts. A data extraction tool was created to help distil relevant descriptive, implementation, measurement and development information for each resource including: the nature of the KT method or tool, relevance for public health, methodological strength, development history, and access issues (e.g., cost, format, language). Tables 1 and 2 provide an overview of the inclusion and data extraction tools, which are available in full on the registry website. To support preparation of consistent syntheses of the extracted information a summary statement template was also developed. Finally, an interactive website with a searchable database was designed and built to house the registry. Over the course of the first 2 years of the project these processes, tools and technology were piloted and refined.

**Table 1:** Questions from registry inclusion-screening tool (Hamilton, ON, Canada, 2011)

1. The resource contains a method or tool
No (stop; exclude)
Yes (continue)
2a. The method/tool is used for knowledge translation (KT)
No (stop; exclude)
Yes (choose those that apply and continue)
Synthesis
Dissemination
Exchange
Application
2b. The method/tool incorporates the following stage of activity for KT (choose those that apply and continue)
Planning
Doing
Evaluating
3. The method/tool is relevant and/or can be adapted to the Canadian public health context.
No (stop; exclude)
Yes (continue)

**Table 2:** Questions from registry data extraction tool (Hamilton, ON, Canada, 2011)

Description
1. Describe the purpose and rationale for the method/tool
2. Briefly describe any theories, models, frameworks, principles and/or philosophies used to develop the method/tool
3. Briefly describe the method/tool (list questions, sections, elements, activities)
4. The method/tool was developed for use in public health contexts (yes or no)
5. The method/tool is transferable to public health contexts (yes or no)
6. Provide a specific example for how the method/tool can be used in public health
Implementation
7. Briefly describe the steps/process for using/implementing the method/tool
8. Who is involved in the delivery and/or administration of the method/tool?
9. Who is involved as participants/respondents of the method/tool?
Evaluation and measurement characteristics
10. Indicate whether the method/tool: has been evaluated, has not been evaluated, evaluation in progress, or information not available
11. Indicate whether the validity properties of the method/tool: meet accepted standards, do not meet accepted standards, have not been tested, testing in progress, information not available, or validity properties not applicable
12. Reliability properties of the method/tool: meet accepted standards, do not meet accepted standards, have not been tested, testing in progress, information not available, or reliability properties not applicable
13. Choose an appropriate methodological rating for the method/tool: strong, moderate, weak, unknown/no evidence, or not applicable
Method/tool development
14. Provide developer information (name, position, organization)
15. List the processes/steps used/taken to develop the method/tool
16. Specify the year the method/tool was first released/made available for use, or when it was first put in practice
17. Provide the contact information available for user support (name, position, organization, address, e-mail, phone)

### Populating the registry

The initial methods and tools considered for inclusion in the registry were drawn from KT resources identified in the NCCMT’s Environmental Scan which examined relevant published and grey literature between 1985 and 2006 (Ciliska et al. 2006). In 2008–2009, project staff conducted a targeted search of over 100 websites and a keyword internet search using Google Scholar, hand searched reference lists and relevant journals, and contacted KT experts for recommendations. On an ongoing basis, potential published and grey literature resources are also identified by key informants, through list-serves, and by registry users requesting resources to address specific needs. The latter includes questions to address practice-based issues brought forward by public health professionals through an online discussion forum hosted by the NCCMT (DialoguePH http://www.nccmt.ca/forum/en/index.html).

In 2011, a more comprehensive search of the published literature was undertaken with a systematic search strategy developed by a health sciences librarian that included six bibliographic databases, used 87 key terms and covered the period from January 2006 to January 2011 (see Table 3). The titles and abstracts of all English language citations were reviewed for relevance by two independent screeners using the inclusion tool. All citations deemed potentially relevant by one or both screeners were retrieved for full-text review. Resources deemed relevant underwent data extraction by two independent reviewers. Agreement was reached through discussion and a third person was consulted to resolve persistent conflicts. Using the extracted data and writing template, one project staff prepares the summary statements. A second reviewer assesses the statements for accuracy, clarity and completeness. Summaries are then translated into French and files in both languages are posted on the registry website.

**Table 3:** Search strategy for methods and tools for knowledge translation in public health (Hamilton, ON, Canada, 2011)

Dates: January 2006 to January 2011					
Databases: CINAHL, Cochrane Library, EMBASE, MEDLINE, Psych Info, Sociological Abstracts					
Search terms (applied to title, abstract and subject headings):					
Methodologies/tools	To facilitate use	Of public health	Interventions	For decision making	By practitioners
Method*	Facilitat*	Public health	Intervention*	Decision*	Practitioner*
Model*	Synthesi*	Community health	Evidence*	Policies	Professional*
Tool*	Promot*	Population health	Practice*	Policy	Provider*
Tool kit*	Access*	Preventi*	Information	Plan	Stakeholder*
Portal*	Utiliz*	Health promotion	Strateg*	Plans	Administrator*
Guide*	Utilis*		Recommendation*	Planning	Policy maker*
Best practice*	Transfer*		Assessment	Priorit*	Policy maker*
Clearinghouse*	Implement*		Program*	Analys*	Health personnel
Framework*	Adopt*		Research	Analyz*	Setting*
Instrument*	Aggregate*		Systematic review*	Evaluat*	Physician*
Knowledge transfer	Analyz*		Literature review*		Decision maker*
Knowledge exchange	Inform		Critical appraisal		Organization*
Knowledge	Influenc*				Organization*
Management	Translat*				Communit*
Knowledge dissemination	Assist				Government*
Knowledge translation	Communicat*				Societ*
Diffusion of innovation	Guide*				Agenc*
Pathway*	Institutionali*				Workforce
Recommendation*	Evaluat*				Nurse*
Knowledge broker*	Disseminat*				Opinion leader*
					Change agent*

The asterisk is used as a truncation symbol to search for variations of the term (e.g., disseminat* would retrieve dissemination, disseminate, disseminating, disseminated)

### Registry evaluation

As part of a broader evaluation of the NCCMT’s website services, in winter 2012, the registry underwent a formative review. Research ethics approval was granted by Hamilton Health Sciences/Faculty of Health Sciences Research Ethics Board (McMaster University). The evaluation used a mixed-methods design with an online, cross-sectional survey and semi-structured telephone interviews with NCCMT users. All registered NCCMT users (*n* = 1,983) were invited to complete an online survey via e-mail. A purposeful sample of 50 NCCMT users was invited to participate in individual telephone interviews. Survey and interview respondents were asked to explore several topics including: their awareness of the registry, accessing registry resources, using these KT methods and tools in practice, and needs for specific types of methods and tools not currently featured in the registry. Evaluation findings have been used to inform refinements to the user interface (e.g., website search and navigation features) and the registry products (e.g., summary statements), and to expand the pool of potential resources for populating the registry.

## Results

### Populating the registry

The initial search activities located 518 possible resources in the published and grey literature, 119 of which were full-text screened for inclusion. Of these, 35 were deemed appropriate for the registry. With 8 summary statements completed, the registry was launched as a publicly accessible feature on the NCCMT’s website in fall 2009. Data extraction continued for the remaining methods and tools located in the initial search and summary statements were posted to the registry as they became available.

From the 2011 comprehensive search of the published literature 42,729 unique citations were identified. A total of 562 citations were retrieved for full-text review, 105 of which were deemed appropriate for the registry and did not overlap with the previously included citations. A priority setting exercise was used to establish the order in which these methods and tools are being processed for inclusion in the database. Precedence is being given to: (1) resources requested by registry users, (2) resources that fill gaps in registry categories (e.g., adapting research evidence, policy development) and (3) KT tools (i.e., practical instruments, surveys, checklists).

As of November 2012, the registry included 133 summary statements: 65 on KT methods and 68 on KT tools. Given efforts to advance the science and practice of KT, most methods and tools in the registry are recent with 62 % of the resources published and/or updated in 2006 or later.

### Registry evaluation

A total of 286 registered NCCMT users (269 English, 17 French) responded to the web-based survey. Descriptive statistics illustrated how survey participants have accessed and used resources in the registry. Eighty-five percent (184/217) of survey respondents were aware of the registry and 92 % (162/177) indicated that they would visit the registry again in the future. Many participants (68 %, 122/179) indicated they have shared methods and tools found on the registry with colleagues and 42 % (73/175) have used a registry resource in their work. The main reasons given for accessing registry resources were to: assist with program planning (62 %, 109/176), share evidence with others (57 %, 100/176), critically appraise research evidence (50 %, 88/176), and support policy development (39 %, 69/176).

Nineteen registry users agreed to participate in individual telephone interviews. Interviews were transcribed, imported into a qualitative data management software program (NVivo) and content analysis was conducted to identify major themes in users’ experiences. Participants thought the registry includes a substantial pool of KT methods and tools; they appreciate that more than one resource is often available for a specific task and that website links are provided to facilitate access to the resources. Some participants considered the summary statement feature a key strength of the registry; they valued having access to comprehensive, useful and up-to-date information on each resource. However, both survey and interview respondents recommended streamlining the format and content of the summary statements and providing real life, practice-based examples to demonstrate how specific methods and tools have been used to support KT efforts in public health contexts.

Evaluation results highlighted the need to improve users’ experiences related to searching for appropriate KT resources. Although most survey respondents (67 %, 120/179) indicated that they were able to access relevant methods and tools in the registry, one-third (33 %, 59/179) were neutral or negative with respect to being able to find resources. Many people (43 %, 24/56) indicated that lack of time prevented effective searching, while others thought their key barriers were related to not understanding how to search (34 %, 19/56) and not knowing what resources were available to be found (27 %, 15/56). Some interview respondents had also encountered challenges when trying to locate relevant resources. Mostly, they reported a lack of results with the search terms they were using; instead of entering the KT keywords (e.g., stakeholder analysis) they were using public health content keywords (e.g., immunization).

### Refining the registry

Acting on evaluation results, the registry indexing system was reorganized to make the types of resources available more explicit; categories and labels were added that resonate with users and are specific to functions and tasks for applying research evidence in public health practice. In addition to tagging methods and tools according to the type of KT activity (synthesis, dissemination, application, exchange) and stage of activity (planning, doing, evaluating), registry entries are now categorized according to: (1) their status as a method or a tool, (2) which step(s) in the evidence-informed public health process the resource supports (i.e., define, search, appraise, synthesize, adapt, implement, evaluate) (National Collaborating Centre for Methods and Tools 2011), and/or (3) their association with particular KT tasks or issues (i.e., program planning, policy development, communication, capacity development, partnerships, networks, equity, theories). Usability testing indicates this new categorization strategy better assists users in finding the KT resources that will meet their needs.

Incorporating user feedback, the summary statements have also been remodeled to enhance understanding and utilization of KT resources. Website links to the specific methods and tools now appear at the front end of the summaries along with links to supplemental materials available to support practical application of the resources. When appropriate, statements describe how the method or tool can be used as a part of the evidence-informed public health process and identify complimentary NCCMT resources that can be accessed to support such activities. In addition, summaries now contain links to other resources in the registry and articulate how the identified methods and tools can be used together to accomplish an overarching goal or task (e.g., combining resources for stakeholder mapping, consensus building and evidence synthesis to support groups interested in developing evidence-based guidance in collaboration with communities, practitioners and decision makers).

Introduced in fall 2011, another feature of the registry website is a user stories section that draws on real life experiences of implementing the KT methods and tools in public health settings. These short narratives are profiled on the registry’s main page and the respective online summary statements. As of November 2012, there were four tools in the registry with a user story and more stories are currently in development. Public health professionals are encouraged to submit their experiences with these and other KT methods and tools to expand this important strategy for dissemination.

### Registry users

The NCCMT’s Registry of Methods and Tools attracts a broad audience. Website statistics monitored by Google Analytics from January 1 to November 30, 2012 indicate almost half of users originate from Canada (43 % of 45,081 visits). Many other visitors are based in the United States (19 %) and the United Kingdom (9 %) with the remaining users located in 188 other countries worldwide. For the first 11 months in 2012, Google Analytics counted 32,945 unique visitors accessing the registry with 84,490 page views. These numbers represent a twofold increase in visits over the 2011 calendar year.

### Promoting the registry

The surge in the number of visits may be explained, in part, by concurrent intensive efforts to populate and promote the registry. The registry was officially launched in June 2011 with webinars and oral presentations attended by target users, via an e-mail blast sent to distributed list-serves and through features in other organizations’ newsletters. Between June 2011 and November 2012, 62 more summary statements were posted and a critical mass of methods and tools have been actively promoted by NCCMT during national conference presentations and internationally accessible webinars.

## Discussion

Across public health systems there are increasing efforts to support and encourage knowledge of, access to and use of research evidence to inform program and policy decisions and a number of research programs have been initiated to study and evaluate what works to promote and achieve evidence-informed public health (e.g., Armstrong et al. 2011; Dobbins et al. 2009a, b; Peirson et al. 2012; Waters et al. 2011). In addition to workforce skills development and organizational change initiatives that focus on building capacity and infrastructure for evidence-informed public health, a variety of web-based resources have been created to provide decision makers with access to information, evidence, guidelines and tools.

For example, there are several online, searchable and continually updated collections that house evidence syntheses on public health research. Within the Cochrane Library there is a dedicated section for several dozen reviews that examine the effects of population-level public health interventions (http://ph.cochrane.org). The Evidence for Policy and Practice Information Centre maintains a database that contains a few thousand systematic and non-systematic reviews of effectiveness in health promotion and public health (http://eppi.ioe.ac.uk/webdatabases/Intro.aspx?ID=2). The Centre for Reviews and Dissemination provides access to an extensive library of systematic reviews on the effects of health care interventions and the delivery and organization of health services, economic evaluations of health care interventions, and health technology assessments; more than 1,300 entries are tagged as public health related (http://www.crd.york.ac.uk/crdweb). Created by the McMaster Health Knowledge Refinery, Public Health + is a database of over 1,200 methodologically sound primary studies and systematic reviews on public health topics, distilled from over 120 health journals, that have been rated as both relevant and newsworthy by volunteer experts (http://www.nccmt.ca/public_health_plus/all/1/list-eng.html). Health-evidence is another web-based resource that provides access to more than 2,700 pre-processed (filtered, quality rated and summarized) reviews of health promotion and public health interventions (http://www.health-evidence.ca).

A number of other online resources have been developed to provide the public health community with information, guides and tools to engage in evidence-informed decision making and program planning. For example, the UK National Health Service (NHS) website contains a section for public health evidence that includes 11 topic pages that provide guidance, implementation tools, case studies and information for the public (http://www.evidence.nhs.uk/nhs-evidence-content/public-health). Also available on the NHS website is the National Institutes of Clinical Excellence (NICE) Pathways information network that brings together all the NICE guidance and related NICE products concerning specific health topics, currently including 38 issues relevant to seven public health categories, and organizes them in a series of interactive flowcharts (http://pathways.nice.org.uk). Supported by the Centers for Disease Control and Prevention in the United States, the Guide to Community Preventive Services provides access to systematic and economic reviews and evidence-based recommendations for effective public health interventions spanning 22 topic areas (http://www.thecommunityguide.org/index.html). The Public Health Agency of Canada’s Canadian Best Practices Portal offers an inventory of policies, programs and interventions for chronic disease prevention and health promotion, and connects users to a broad range of products and other websites that: provide information and tools for evidence-informed public health and program planning; contain public health related policy documents, resources and instruments; and provide access to surveillance data, intervention strategies, systematic reviews and practice guidelines for ten public health topic areas (http://cbpp-pcpe.phac-aspc.gc.ca).

Notwithstanding the important contributions of these and other portals and databases for enhancing access to public health research and topic-specific information and resources, a critical gap in the evidence-informed public health landscape was recognized by the architects of the National Collaborating Centres and the public health community (Ciliska et al. 2006; Medlar et al. 2006). There was no existing resource, in Canada or elsewhere, that was dedicated to identifying KT methods and tools relevant for public health contexts and that facilitated quick and easy access to these resources by decision makers. To our knowledge, the NCCMT’s Registry of KT Methods and Tools remains unique in its goals, service and products. At present, the registry only includes methods and tools that are available in English and that are applicable to Canadian public health contexts, although most resources are likely transferable to public health settings in other developed countries. These limitations mean the contents of the registry may not be sufficient to meet the needs of public health decision makers in non-English speaking parts of the world and in developing nations where public health systems may be encountering unique KT challenges related to their own development or to differences in population-based health priorities, technology, and so on. However, the registry’s summary statements and companion materials provide all users with detailed information on which to base decisions about the applicability and transferability of the KT methods and tools for their specific needs and contexts.

## Conclusion

After 5 years of planning, development and implementation, the NCCMT’s Registry of Methods and Tools is an operational, interactive database populated with over 130 resources (with many more in the queue) to support KT activities in public health. Evaluation results suggest that the registry is a valued addition to the complement of resources currently available to support evidence-informed public health. Recent interest in advancing the science and practice of developing and implementing methods and tools for KT suggests there will be a substantial pool of resources for continuing to populate the NCCMT’s Registry and sustaining this database as a key resource for supporting the complex, dynamic and critical work of public health. As part of the ongoing efforts to build the capacity and infrastructure needed to support evidence-informed public health, the registry facilitates access to KT resources that can help decision makers integrate research knowledge into practice. The increasing number of visitors to the registry and the expanding interest in and use of KT methods and tools reinforces the relevance of this unique and practical resource for public health decision makers worldwide.
